# Supplementary material for: Early excitatory-inhibitory cortical modifications following skill learning are associated with motor memory consolidation and plasticity overnight
Source: Nat Commun. 2024 Jan 30;15:906. doi: 10.1038/s41467-024-44979-9 (PMC10828487; doi:10.1038/s41467-024-44979-9)
Supplement: Supplementary file 1 — Supplementary Information [file 41467_2024_44979_MOESM1_ESM.pdf]

Early excitatory-inhibitory cortical modifications following skill learning associate with motor memory consolidation and plasticity overnight

Supplementary Information

Supplementary Note 1

MRS quality metrics

The MRS quality metrics are presented in **Supplementary Table 1**. Out of 216 MRS spectra across all participants in the Learning group (36 participants x 6 MRS runs), 5 spectra (across 3 participants) were not included in the final data analyses due to low SNR (i.e., <30) or contaminated spectra, and 2 other spectra from an additional participant were not acquired due to time limitation in the scanner.

Out of 126 MRS spectra across all participants in the control group (21 participants x 6 runs), 2 spectra from one participant were excluded due to water linewidth > 15 Hz, and an additional spectrum from another participant was excluded due to SNR < 20. Four additional individual GABA measurements from four different participants were excluded for being more extreme than 3 SD below the mean GABA concentrations.

Main effect of Run ($F(5,53.01)=3.94$, $p=.004$), but not Run x Group interaction ($F(5,53.01)=0.95$, $p=.454$) was found for SNR across all MRS measurements. Post-hoc pairwise comparisons showed that the POST 20 min, POST 25 min, and POST 30 min runs had lower SNR compared to the PRE run ($pFDR<0.05$), however the mean absolute difference was generally small (ranging between 1.87-2.17 SNR units). The same pattern was found in terms of water linewidth with main effect of Run ($F(5,46.43)=3.82$, $p=.006$) and no Run x Group interaction ($F(5,46.01)=1.63$, $p=.172$). Post-hoc pairwise comparisons showed that the POST 20 min, POST 25 min, and POST 30 min runs had higher water linewidth compared to the PRE run ($pFDR<0.05$), however the mean absolute difference was also generally small (ranging between 0.396-0.653 Hz). No main effect of Run ($F(1,55)=0.57$, $p=.455$) nor Run x Group interaction ($F(1,55)=1.93$, $p=.171$) were found for GM fraction, WM fraction (Run main effect: ($F(1,55)=0.49$, $p=.485$); Run x Group interaction: ($F(1,55)=2.26$, $p=.139$)), or CSF fraction (Run main effect: ($F(1,55)=0.04$, $p=.836$); Run x Group interaction: ($F(1,55)=0.98$, $p=.326$)) across the two scanning sessions overnight.

Supplementary Table 1. MRS quality metrics (Mean±SD)

MRS run	Water FWHM (Hz)	SNR	% CRLB (GABA)	Voxel GM fraction	Voxel WM fraction	Voxel CSF fraction
Learning						
Pre-learning	10.67±1.18	59.06±5.67	29.33±8.3	0.373±0.03	0.575±0.05	0.052±0.03
Post 5 min	10.79±1.08	58.83±5.68	33.54±15.3	0.373±0.03	0.575±0.05	0.052±0.03
Post 20 min	10.67±1.09	58.49±5.47	28.51±7.2	0.373±0.03	0.575±0.05	0.052±0.03
Post 25 min	10.81±1.22	57.28±6.14	32.90±12.6	0.373±0.03	0.575±0.05	0.052±0.03
Post 30 min	10.94±1.18	58.1±5.11	29.06±5.9	0.373±0.03	0.575±0.05	0.052±0.03
Overnight	10.58±1.09	60.83±5.78	29.86±9.7	0.370±0.03	0.579±0.05	0.050±0.03
Rest						
Pre-learning	10.61±1.16	57.48±8.37	33.52±19.1	0.342±0.04	0.613±0.05	0.046±0.03
Post 5 min	10.97±1.09	57.05±7.45	30.38±7.0	0.342±0.04	0.613±0.05	0.046±0.03
Post 20 min	11.39±1.42	54.24±8.56	31.15±9.05	0.342±0.04	0.613±0.05	0.046±0.03
Post 25 min	11.43±1.23	54.95±6.55	32.53±15.6	0.342±0.04	0.613±0.05	0.046±0.03
Post 30 min	11.74±1.59	55.05±7.61	30.72±8.4	0.342±0.04	0.613±0.05	0.046±0.03
Overnight	10.97±1.16	57.52±7.63	29.10±10.0	0.351±0.05	0.601±0.06	0.048±0.02

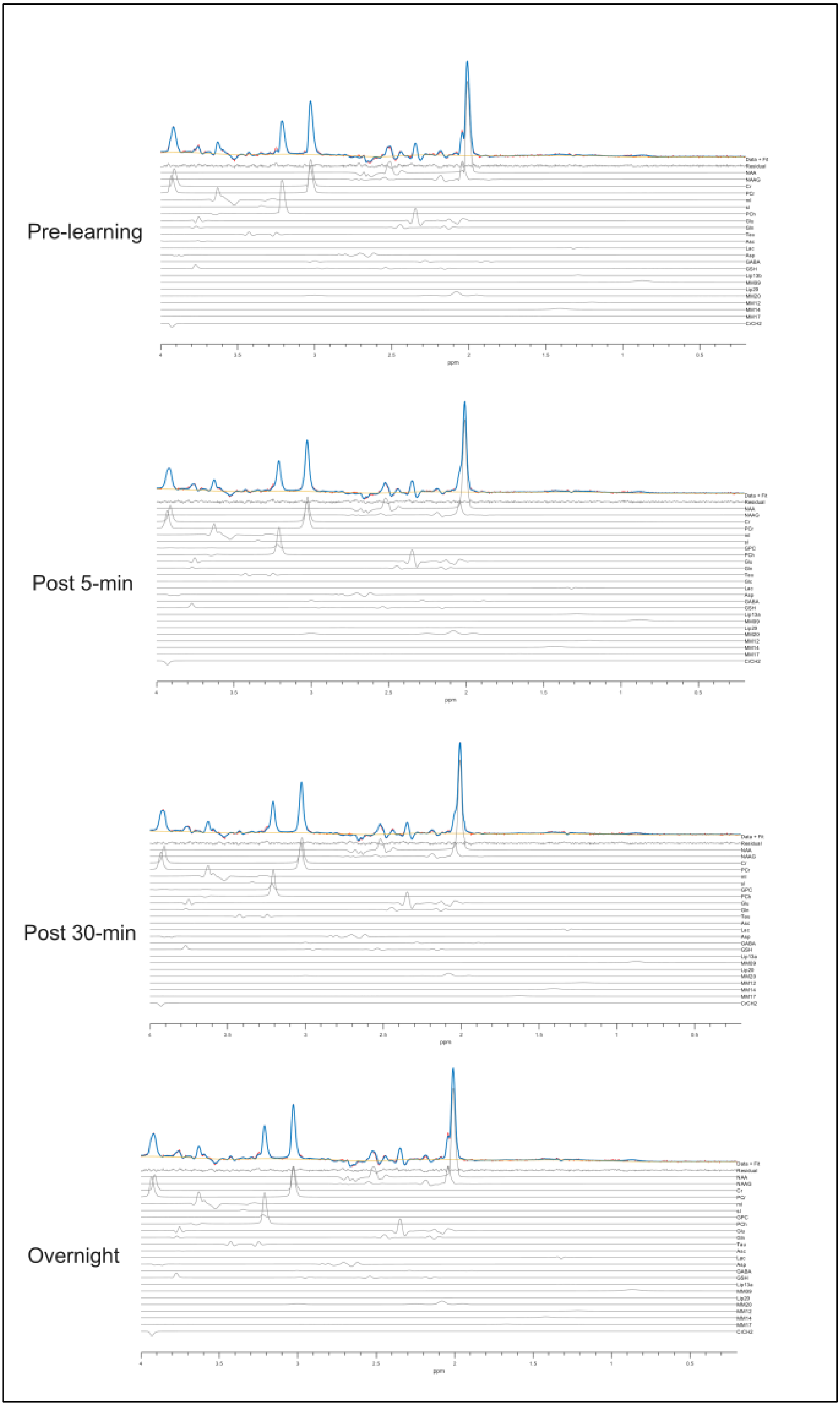

Supplementary Figure 1. Representative examples of LCModel fitting from one participant acquired as a function of time

Supplementary Table 2. Absolute concentrations of Glu and GABA before and after learning or resting control condition.

MRS run	Pre-learning	Post 5 min	Post 20 min	Post 25 min	Post 30 min	Overnight
Learning						
Glu (mM)	7.89±.74	8.02±.74	8.02±.69	8.05±.80	8.07±.68	8.03±.59
GABA (mM)	.78±.19	.75±.26	.82±.19	.76±.23	.80±.16	.77±.20
Rest						
Glu (mM)	7.75±.91	8.08±.95	7.72±1.02	7.87±.96	7.66±1.01	7.77±1.03
GABA (mM)	.76±.22	.78±.17	.78±.18	.80±.24	.75±.18	.82±.23

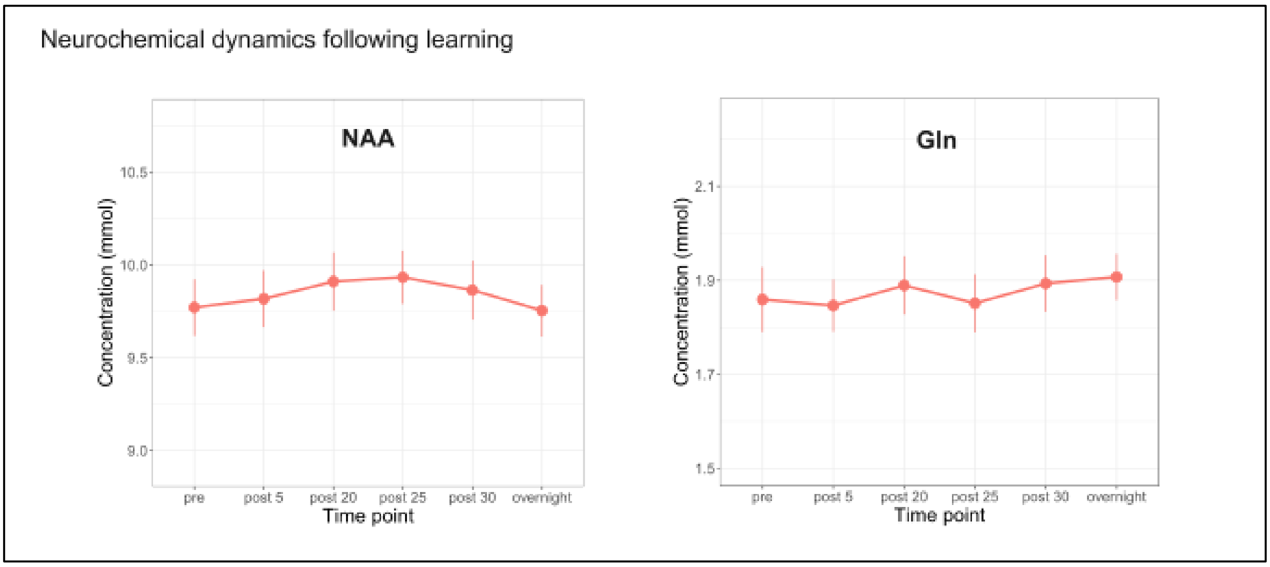

Supplementary Figure 2. Neurochemical dynamics of NAA and Gln following learning. No significant changes were observed for each of the metabolites following the MSL task. Source data are provided as a Source Data file.

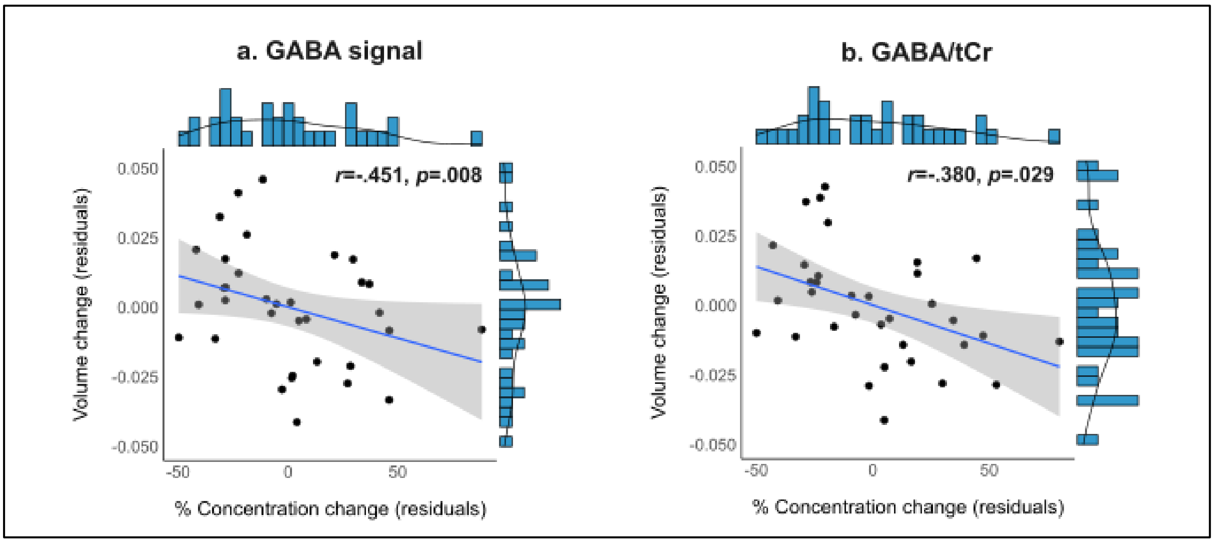

Supplementary Figure 3. Correlation between GABA changes 30 minutes following learning and overnight changes in M1 GM volume, when GABA expressed as the raw signal (A) or referenced to total creatine (B). Source data are provided as a Source Data file.

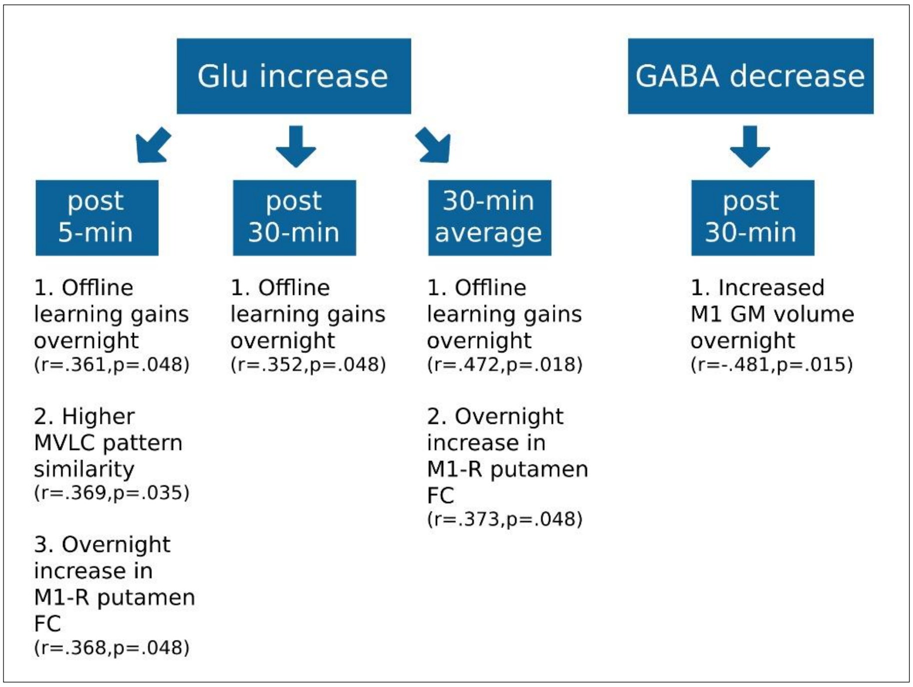

Supplementary Figure 4. Summary of the significant associations observed in the experiment between post-learning Glu and GABA changes and neuro-behavioral correlates of motor memory consolidation. FC = functional connectivity; GM = grey matter; R = right
