# Supplementary material for: Early excitatory-inhibitory cortical modifications following skill learning are associated with motor memory consolidation and plasticity overnight
Source: Nat Commun. 2024 Jan 30;15:906. doi: 10.1038/s41467-024-44979-9 (PMC10828487; doi:10.1038/s41467-024-44979-9)
Supplement: Supplementary file 3 — Reporting Summary [file 41467_2024_44979_MOESM3_ESM.pdf]

nature portfolio

Corresponding author(s): Assaf Tal

Last updated by author(s): Nov 15, 2023

Reporting Summary

Nature Portfolio wishes to improve the reproducibility of the work that we publish. This form provides structure for consistency and transparency in reporting. For further information on Nature Portfolio policies, see our Editorial Policies and the Editorial Policy Checklist.

Statistics

For all statistical analyses, confirm that the following items are present in the figure legend, table legend, main text, or Methods section.

n/a	Confirmed	
☐	☒	The exact sample size (n) for each experimental group/condition, given as a discrete number and unit of measurement
☐	☒	A statement on whether measurements were taken from distinct samples or whether the same sample was measured repeatedly
☐	☒	The statistical test(s) used AND whether they are one- or two-sided<br>*Only common tests should be described solely by name; describe more complex techniques in the Methods section.*
☐	☒	A description of all covariates tested
☐	☒	A description of any assumptions or corrections, such as tests of normality and adjustment for multiple comparisons
☐	☒	A full description of the statistical parameters including central tendency (e.g. means) or other basic estimates (e.g. regression coefficient) AND variation (e.g. standard deviation) or associated estimates of uncertainty (e.g. confidence intervals)
☐	☒	For null hypothesis testing, the test statistic (e.g. F, t, r) with confidence intervals, effect sizes, degrees of freedom and P value noted<br>*Give P values as exact values whenever suitable.*
☒	☐	For Bayesian analysis, information on the choice of priors and Markov chain Monte Carlo settings
☒	☐	For hierarchical and complex designs, identification of the appropriate level for tests and full reporting of outcomes
☐	☒	Estimates of effect sizes (e.g. Cohen's d, Pearson's r), indicating how they were calculated

Our web collection on statistics for biologists contains articles on many of the points above.

Software and code

Policy information about availability of computer code

Data collection	Behavioral data were collected using MATLAB 21a and the Psychophysics Toolbox v3
Data analysis	Data analyses were performed with MATALB 21a, R 4.1.2, FSL 6.05, and the Conn Toolbox 21a

For manuscripts utilizing custom algorithms or software that are central to the research but not yet described in published literature, software must be made available to editors and reviewers. We strongly encourage code deposition in a community repository (e.g. GitHub). See the Nature Portfolio guidelines for submitting code & software for further information.

Data

Policy information about availability of data

All manuscripts must include a data availability statement. This statement should provide the following information, where applicable:

- Accession codes, unique identifiers, or web links for publicly available datasets
- A description of any restrictions on data availability
- For clinical datasets or third party data, please ensure that the statement adheres to our policy

Due to medical confidentiality of participants' data, the raw imaging data cannot be uploaded to a public repository. The unidentified data spreadsheets generated and analyzed in the current study are available from the corresponding author on request.

Research involving human participants, their data, or biological material

Policy information about studies with human participants or human data. See also policy information about sex, gender (identity/presentation), and sexual orientation and race, ethnicity and racism.

Reporting on sex and gender	The distribution of the sexes within the study groups are reported (based on self-report of the participants)
Reporting on race, ethnicity, or other socially relevant groupings	NA
Population characteristics	57 healthy right-handed young adults (age 27.5 +/- 5.2 years, 24 females)
Recruitment	Participants were recruited using online ads and word-of-mouth. Group allocation was not blind to the investigators since the Control Group was only recruited during revision following a reviewer's request. Participants in the Control Group were not aware of the other group, and the fact that they were serving as a control setting in a learning and memory study. Given the recruitment method and the strict safety inclusion criteria of the 7T MRI scanning there may be a selection bias, which we do not expect to influence motor learning.
Ethics oversight	All participants provided written informed consent to participate in the study procedures, approved by the Wolfson Medical Center's Helsinki Committee (Holon, Israel), and the Institutional Review Board (IRB) of the Weizmann Institute of Science (Rehovot, Israel)

Note that full information on the approval of the study protocol must also be provided in the manuscript.

Field-specific reporting

Please select the one below that is the best fit for your research. If you are not sure, read the appropriate sections before making your selection.

☒ Life sciences ☐ Behavioural & social sciences ☐ Ecological, evolutionary & environmental sciences

For a reference copy of the document with all sections, see nature.com/documents/nr-reporting-summary-flat.pdf

Life sciences study design

All studies must disclose on these points even when the disclosure is negative.

Sample size	57 healthy young adults. Sample size calculation was not performed in advance, however the study sample size adhere and even exceeds current studies utilizing 7T MRI scanning in which the sample sizes are generally limited due to the strict inclusion criteria of 7T safety guidelines.
Data exclusions	Participants MRS measurements were excluded based on previously published recommended criteria which are detailed in the Methods section of the manuscript. The exclusion criteria were pre-established.
Replication	Replication was not performed due to the inability to recruit a matching group of participants due to time limitation of the study project and the strict exclusion criteria of the 7T MRI system.
Randomization	Randomization was not performed as part of the group allocation since the Control Group was only recruited during revision following a reviewer's request. The groups were not statistically different in basic demographic characteristics such Age and Sex.
Blinding	Blinding of the investigators was not possible in the current study since the Control Group was only recruited during revision following a reviewer's request. The Control Group participants were not aware of the other experimental group and that they were participating in a learning and memory experiment.

Reporting for specific materials, systems and methods

We require information from authors about some types of materials, experimental systems and methods used in many studies. Here, indicate whether each material, system or method listed is relevant to your study. If you are not sure if a list item applies to your research, read the appropriate section before selecting a response.

Materials & experimental systems

n/a	Involved in the study
☐	☐ Antibodies
☐	☐ Eukaryotic cell lines
☐	☐ Palaeontology and archaeology
☐	☐ Animals and other organisms
☐	☐ Clinical data
☐	☐ Dual use research of concern
☐	☐ Plants

Methods

n/a	Involved in the study
☐	☐ ChIP-seq
☐	☐ Flow cytometry
☐	☐ MRI-based neuroimaging

Antibodies

Antibodies used — *Describe all antibodies used in the study; as applicable, provide supplier name, catalog number, clone name, and lot number.*

Validation — *Describe the validation of each primary antibody for the species and application, noting any validation statements on the manufacturer's website, relevant citations, antibody profiles in online databases, or data provided in the manuscript.*

Eukaryotic cell lines

Policy information about cell lines and Sex and Gender in Research

Cell line source(s) — *State the source of each cell line used and the sex of all primary cell lines and cells derived from human participants or vertebrate models.*

Authentication — *Describe the authentication procedures for each cell line used OR declare that none of the cell lines used were authenticated.*

Mycoplasma contamination — *Confirm that all cell lines tested negative for mycoplasma contamination OR describe the results of the testing for mycoplasma contamination OR declare that the cell lines were not tested for mycoplasma contamination.*

Commonly misidentified lines (See ICLAC register) — *Name any commonly misidentified cell lines used in the study and provide a rationale for their use.*

Palaeontology and Archaeology

Specimen provenance — *Provide provenance information for specimens and describe permits that were obtained for the work (including the name of the issuing authority, the date of issue, and any identifying information). Permits should encompass collection and, where applicable, export.*

Specimen deposition — *Indicate where the specimens have been deposited to permit free access by other researchers.*

Dating methods — *If new dates are provided, describe how they were obtained (e.g. collection, storage, sample pretreatment and measurement), where they were obtained (i.e. lab name), the calibration program and the protocol for quality assurance OR state that no new dates are provided.*

☐ Tick this box to confirm that the raw and calibrated dates are available in the paper or in Supplementary Information.

Ethics oversight — *Identify the organization(s) that approved or provided guidance on the study protocol, OR state that no ethical approval or guidance was required and explain why not.*

Note that full information on the approval of the study protocol must also be provided in the manuscript.

Animals and other research organisms

Policy information about studies involving animals; ARRIVE guidelines recommended for reporting animal research, and Sex and Gender in Research

Laboratory animals — *For laboratory animals, report species, strain and age OR state that the study did not involve laboratory animals.*

Wild animals — *Provide details on animals observed in or captured in the field; report species and age where possible. Describe how animals were caught and transported and what happened to captive animals after the study (if killed, explain why and describe method; if released, say where and when) OR state that the study did not involve wild animals.*

Reporting on sex — *Indicate if findings apply to only one sex; describe whether sex was considered in study design, methods used for assigning sex. Provide data disaggregated for sex where this information has been collected in the source data as appropriate; provide overall*

	numbers in this Reporting Summary. Please state if this information has not been collected. Report sex-based analyses where performed, justify reasons for lack of sex-based analysis.
Field-collected samples	*For laboratory work with field-collected samples, describe all relevant parameters such as housing, maintenance, temperature, photoperiod and end-of-experiment protocol OR state that the study did not involve samples collected from the field.*
Ethics oversight	*Identify the organization(s) that approved or provided guidance on the study protocol, OR state that no ethical approval or guidance was required and explain why not.*

Note that full information on the approval of the study protocol must also be provided in the manuscript.

Clinical data

Policy information about clinical studies
All manuscripts should comply with the ICMJE guidelines for publication of clinical research and a completed CONSORT checklist must be included with all submissions.

Clinical trial registration	*Provide the trial registration number from ClinicalTrials.gov or an equivalent agency.*
Study protocol	*Note where the full trial protocol can be accessed OR if not available, explain why.*
Data collection	*Describe the settings and locales of data collection, noting the time periods of recruitment and data collection.*
Outcomes	*Describe how you pre-defined primary and secondary outcome measures and how you assessed these measures.*

Dual use research of concern

Policy information about dual use research of concern

Hazards

Could the accidental, deliberate or reckless misuse of agents or technologies generated in the work, or the application of information presented in the manuscript, pose a threat to:

No	Yes	
☐	☐	Public health
☐	☐	National security
☐	☐	Crops and/or livestock
☐	☐	Ecosystems
☐	☐	Any other significant area

Experiments of concern

Does the work involve any of these experiments of concern:

No	Yes	
☐	☐	Demonstrate how to render a vaccine ineffective
☐	☐	Confer resistance to therapeutically useful antibiotics or antiviral agents
☐	☐	Enhance the virulence of a pathogen or render a nonpathogen virulent
☐	☐	Increase transmissibility of a pathogen
☐	☐	Alter the host range of a pathogen
☐	☐	Enable evasion of diagnostic/detection modalities
☐	☐	Enable the weaponization of a biological agent or toxin
☐	☐	Any other potentially harmful combination of experiments and agents

Plants

Seed stocks | *Report on the source of all seed stocks or other plant material used. If applicable, state the seed stock centre and catalogue number. If plant specimens were collected from the field, describe the collection location, date and sampling procedures.*

Novel plant genotypes | *Describe the methods by which all novel plant genotypes were produced. This includes those generated by transgenic approaches, gene editing, chemical/radiation-based mutagenesis and hybridization. For transgenic lines, describe the transformation method, the number of independent lines analyzed and the generation upon which experiments were performed. For gene-edited lines, describe the editor used, the endogenous sequence targeted for editing, the targeting guide RNA sequence (if applicable) and how the editor was applied.*

Authentication | *Describe any authentication procedures for each seed stock used or novel genotype generated. Describe any experiments used to assess the effect of a mutation and, where applicable, how potential secondary effects (e.g. second site T-DNA insertions, mosiacism, off-target gene editing) were examined.*

ChIP-seq

Data deposition

☐ Confirm that both raw and final processed data have been deposited in a public database such as GEO.

☐ Confirm that you have deposited or provided access to graph files (e.g. BED files) for the called peaks.

Data access links
May remain private before publication. | *For "Initial submission" or "Revised version" documents, provide reviewer access links. For your "Final submission" document, provide a link to the deposited data.*

Files in database submission | *Provide a list of all files available in the database submission.*

Genome browser session
(e.g. UCSC) | *Provide a link to an anonymized genome browser session for "Initial submission" and "Revised version" documents only, to enable peer review. Write "no longer applicable" for "Final submission" documents.*

Methodology

Replicates | *Describe the experimental replicates, specifying number, type and replicate agreement.*

Sequencing depth | *Describe the sequencing depth for each experiment, providing the total number of reads, uniquely mapped reads, length of reads and whether they were paired- or single-end.*

Antibodies | *Describe the antibodies used for the ChIP-seq experiments; as applicable, provide supplier name, catalog number, clone name, and lot number.*

Peak calling parameters | *Specify the command line program and parameters used for read mapping and peak calling, including the ChIP, control and index files used.*

Data quality | *Describe the methods used to ensure data quality in full detail, including how many peaks are at FDR 5% and above 5-fold enrichment.*

Software | *Describe the software used to collect and analyze the ChIP-seq data. For custom code that has been deposited into a community repository, provide accession details.*

Flow Cytometry

Plots

Confirm that:

☐ The axis labels state the marker and fluorochrome used (e.g. CD4-FITC).

☐ The axis scales are clearly visible. Include numbers along axes only for bottom left plot of group (a 'group' is an analysis of identical markers).

☐ All plots are contour plots with outliers or pseudocolor plots.

☐ A numerical value for number of cells or percentage (with statistics) is provided.

Methodology

Sample preparation | *Describe the sample preparation, detailing the biological source of the cells and any tissue processing steps used.*

Instrument | *Identify the instrument used for data collection, specifying make and model number.*

Software | *Describe the software used to collect and analyze the flow cytometry data. For custom code that has been deposited into a community repository, provide accession details.*

Cell population abundance | *Describe the abundance of the relevant cell populations within post-sort fractions, providing details on the purity of the samples and how it was determined.*

Gating strategy | *Describe the gating strategy used for all relevant experiments, specifying the preliminary FSC/SSC gates of the starting cell population, indicating where boundaries between "positive" and "negative" staining cell populations are defined.*

☐ Tick this box to confirm that a figure exemplifying the gating strategy is provided in the Supplementary Information.

Magnetic resonance imaging

Experimental design

Design type	Multimodal - structural MRI, functional MRI, MR Spectroscopy
Design specifications	The Learning Group underwent the following MR runs in the first day: T1 MP2RAGE, resting MRS, resting fMRI-EPI, task fMRI-EPI (12 blocks of 30 s separated by 30 s resting fixation blocks), resting MRS, resting fMRI-EPI, resting MRS, resting MRS, resting MRS. The Control group underwent the same protocol with a resting fMRI instead of the task. On the second day both groups underwent T1 MP2RAGE, resting MRS, and resting fMRI-EPI. The Learning Group also underwent one block of task fMRI-EPI run.
Behavioral performance measures	Number of correct sequences tapped during the learning task's blocks.

Acquisition

Imaging type(s)	Structural, functional (resting-state and task), and MRS
Field strength	7T
Sequence & imaging parameters	MP2RAGE - TR/TE/TI1/TI2=4460/2.19/1000/3200 ms, 1mm isotropic voxels<br>SemiLASER - TR/TE=7000/80 ms, NEX=36<br>GE-EPI sequence based on the Human Connectome Project 7T protocol
Area of acquisition	Structural and functional - whole brain<br>MRS - primary motor cortex (M1)

Diffusion MRI ☐ Used ☒ Not used

Preprocessing

Preprocessing software	FSL 6.05 and Conn Toolbox 21a
Normalization	Registration of high resolution structural to standard space was carried out using FSL FLIRT, and then further refined using FSL FNIRT non-linear registraton
Normalization template	MNI-152
Noise and artifact removal	ICA-AROMA and regressing out the signal ofthe first component of the CSF extracted with the CompCor method implemented in the Conn Toolbox
Volume censoring	No

Statistical modeling & inference

Model type and settings	GLM for activation analysis in the task fMRI, ROI-to-ROI correlation analysis for resting-state functional connectivity, FSL-VBM processing for structural analysis
Effect(s) tested	*Define precise effect in terms of the task or stimulus conditions instead of psychological concepts and indicate whether ANOVA or factorial designs were used.*

Specify type of analysis: ☐ Whole brain ☐ ROI-based ☒ Both

Anatomical location(s) | M1 ROI was initially defined as the caudal part of the precentral gyrus (the anterior wall of the central sulcus, which directly controls the fingers (Witham et al., 2016; Dubbioso et al., 2021). Then, he final M1 ROI was defined as the set of voxels within this region that demonstrated statistically significant activation in both day 1 and day 2. ROIs of right and left putamen were defined in the same way, based on subcortical segmentation of the MNI-152 template. The ROI of the PCC was extracted from the Harvard-Oxford Atlas, thresholded at 30% and binarized.

Statistic type for inference

(See Eklund et al. 2016)

GLM beta value for task-based activation analysis, Pearson r-to-z transformation in funcntional connectivity analysis

Correction

Cluster correction for whole brain maps of activation during the MSL task. Multiple comparisons correction was spatially restricted to include only grey matter voxels using MNI-152 grey matter mask.

Models & analysis

n/a	Involved in the study
☐	☒ Functional and/or effective connectivity
☒	☐ Graph analysis
☒	☐ Multivariate modeling or predictive analysis

Functional and/or effective connectivity

ROI-to-ROI analysis
